# Facile Synthesis of Fluorinated Polysilazanes and Their Durable Icephobicity on Rough Al Surfaces

**DOI:** 10.3390/polym14020330

**Published:** 2022-01-14

**Authors:** Tien N. H. Lo, Sung Woo Hong, Ha Soo Hwang, In Park

**Affiliations:** 1 Research Institute of Clean Manufacturing System, Korea Institute of Industrial Technology (KITECH), 89 Yangdaegiro-gil, Ipjang-myeon, Cheonan 31056, Korea; htien@kitech.re.kr (T.N.H.L.); swhong@kitech.re.kr (S.W.H.); 2 R&D Center, OomphChem Inc., 1223-24 Cheonan-daero, Seobuk-gu, Cheonan 31080, Korea; 3 KITECH School, University of Science and Technology (UST), 176 Gajeong-dong, Yuseong-gu, Daejeon 34113, Korea

**Keywords:** fluorinated polysilazane, superhydrophobic, anti-icing, icephobic, ice adhesion, freezing delay

## Abstract

Superhydrophobic Al surfaces with excellent durability and anti-icing properties were fabricated by coating dual-scale rough Al substrates with fluorinated polysilazane (FPSZ). Flat Al plates were etched using an acidic solution, followed by immersion in boiling water to generate hierarchical micro-nano structures on their surfaces. The FPSZ coatings were synthesized by grafting 1H,1H,2H,2H-perfluorodecyltrimethoxysilane (FAS-17), a fluoroalkyl silane), onto methylpolysilazane, an organopolysilazane (OPSZ) backbone. The high water contact angle (175°) and low sliding angle (1.6°) of the FPSZ-coated sample with an FAS-17 content of 17.3 wt% promoted the efficient removal of a frozen ice column with a low ice adhesion strength of 78 kPa at −20.0 °C (70% relative humidity), which was 4.3 times smaller than that of an OPSZ-coated surface. The FPSZ-coated Al surface suppressed ice nucleation, leading to a decrease in ice nucleation temperature from −19.5 to −21.9 °C and a delay in freezing time from 334 to 4914 s at −19.0 °C compared with the OPSZ-coated Al surface. Moreover, after 40 icing–melting cycles the freezing temperature of a water droplet on the FPSZ-coated Al surface remained unchanged, whereas that on the FAS-17-coated Al surface increased from −22.3 to −20.7 °C. Therefore, the durability of the polymeric FPSZ coating was superior to that of the FAS-17 monolayer coating.

## 1. Introduction

Typically, ice forms and accumulates on solid surfaces in winter, causing energy loss and significant damage to various industrial equipment, including refrigeration and heat pump systems [1], aircrafts [2], ships [3], power lines [4], and wind turbines [5]. Icephobicity consists of two properties: anti-icing and deicing (or defrosting) [6]. Anti-icing is related to a delay in ice growth rate, whereas deicing is associated with the adhesion strength of ice to solid surfaces. In addition to these properties, durability should be evaluated because icephobic surfaces are rough; therefore, they can be easily damaged by multiple icing–melting cycles.

Recently, the development of superhydrophobic surfaces with anti-icing properties has become a popular topic and attracted the attention of many researchers worldwide [6,7,8,9,10]. Because ice presents low adhesivity to superhydrophobic surfaces, it can be easily removed by delaying its formation or using natural forces, such as wind, gravity, and vibration [11]. However, recently several researchers have demonstrated that superhydrophobic surfaces do not always exhibit excellent anti-icing properties, and some exhibit poor icephobicity under high humidity conditions and after multiple icing–melting cycles [12,13,14]. This was attributed to moisture entering and condensing inside the well-developed pores on the superhydrophobic surfaces and the weak mechanical properties of the hierarchical nanostructures [15,16,17]. Therefore, researchers should focus on improving durability by preventing moisture condensation inside pores and reinforcing nanostructures to obtain superhydrophobic surfaces with excellent anti-icing performance.

Aluminum (Al) is widely used in various industries; therefore, numerous studies on the anti-icing properties of Al surfaces have been conducted over the past decade. For example, Shen et al. fabricated a superhydrophobic surface by coating heptadecafluorodecyl tripropoxysilane on an Al surface with hierarchical micro-nanostructure. The superhydrophobic surface presented a delayed freezing time of 769 s compared with 13.5 s for the bare Al surface. Moreover, the ice adhesion strength of the coated Al surface (75 kPa) was 90% lower than that of the untreated Al surface (700 kPa) [18]. Wang et al. reported the excellent anti-icing properties of a nano-microstructured Al surface coated with 1H,1H,2H,2H-perfluorodecyltriethoxysilane. Although ice adhesion is significantly affected by humidity, the effect of humidity on ice adhesion strength has not been thoroughly evaluated yet [19]. Xie et al. indicated that the freezing delay time of the superhydrophobic surface of an 1H,1H,2H,2H-perfluorodecyltriethoxysilane-coated Al substrate was 50 times longer than that of an untreated Al substrate at −10 °C [20]. However, the considerable increase in ice adhesion strength from 45 to 230 kPa after 20 icing–deicing cycles indicated that the durability of the surface deteriorated after numerous icing–deicing cycles. In addition, improving the anti-icing durability of superhydrophobic surfaces with hierarchical nanostructures remains a challenging task. To effectively use a superhydrophobic Al substrate as an anti-icing material, it is critical to simultaneously improve its robustness and durability. Therefore, a more systematic study on the deicing performance of superhydrophobic Al surfaces should be performed, and new coating materials should be developed to improve the robustness and durability of superhydrophobic Al surfaces.

In our previous study, we synthesized semi-fluorinated polysilazanes via ammonolysis and coated them on Al surfaces with hierarchical nanostructures to prepare icephobic surfaces [21]. The initial ice adhesion strength and that measured after 40 icing–melting cycles were 73 and 131 kPa, respectively, demonstrating the feasibility of semi-fluorinated polysilazane as an anti-icing coating material. Ammonolysis is a useful method for manufacturing fluorine-based polysilazanes; however, a special cabinet and high safety regulations are required to use ammonia gas, which is challenging to handle. In this regard, the use of commercially available organopolysilazanes (OPSZs) should be considered. Recently, several studies have suggested various methods to compensate for the inadequate performance of OPSZs by introducing functional groups into the backbone of Durazane™. For example, Furtat et al. introduced trifluoroethoxy functional groups in the side chain of Durazane 1800 using a catalytic reaction. This led to an increase in the contact angles (CAs) of water and oil by 15% and 40%, respectively, owing to the low surface energy of the fluorinated functional group. In addition, the coated surface exhibited remarkable resistance to acids and bases. However, alkoxy group can be hydrolyzed by moisture, and the Si–O–CH_2_–CF_3_ moieties in the polymer structure can be easily decomposed into trifluoroethanol [22]. Nguyen et al. first introduced a vinyl functional group at the end of monomethoxy poly(ethylene oxide) glycol (MPEG), followed by hydrosilylation with the Si–H functional groups in the backbone of Durazane 1500 [23]. The antifouling performance of the OPSZ–graft–MPEG-coated surface was superior to that of the OPSZ-coated surface. However, in the presence of a base, such as ammonia, the activity of the Karstedt catalyst used for hydrosilylation was significantly lower [24]. Because of this effect, a larger amount of catalyst and a longer reaction time compared with those required for a conventional hydrosilylation reaction were required. Therefore, easier and more effective methods for the modification of OPSZs with fluorinated functional groups should be developed.

In this study, partially fluorinated polysilazanes were easily synthesized by grafting fluorinated alkoxysilanes onto the backbone of Durazane™ via hydrolysis and condensation reactions. Fluorinated polysilazanes (FPSZs) were coated on Al surfaces with the hierarchical structure prepared via chemical etching and hot water treatment. The icephobicity and durability of the coated superhydrophobic surfaces were evaluated, and the effect of the fluoroalkyl content on icephobicity was evaluated by analyzing the wettability, ice adhesion, and ice nucleation delay. The new FPSZ-based coating materials promoted the long-term stability of the anti-icing properties of the Al surfaces, which was evaluated using mechanical erosion (sand particle impact), ice adhesion after multiple icing–melting cycle tests, and ice nucleation temperature experiments.

## 2. Materials and Methods

### 2.1. Materials

**Scheme 1:**
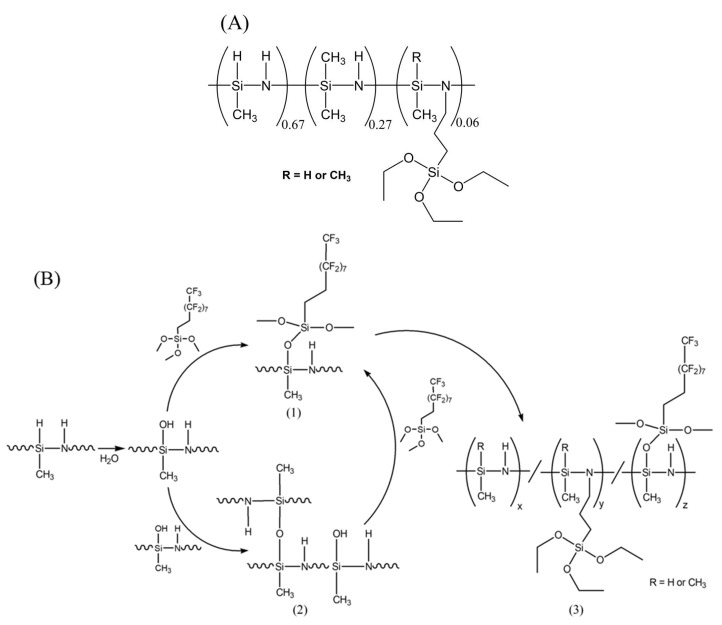
(**A**) Molecular structure of OPSZ. (**B**) Primary reactions between the OPSZ backbone and FAS-17 for FPSZ synthesis.

Durazane™ 1500RC (Merck, Germany), consisting of 3-aminopropyltriethoxysilane-substituted polymethyl-(hydro)/polymethylsilazane, was used as the OPSZ precursor (M_w_ = 1745, PDI = 22), and its molecular structure is illustrated in Scheme 1A. 1H,1H,2H,2H-Perfluorodecyltrimethoxysilane (FAS-17) was obtained from Sooyang Chemtec (Gamgok-myeon, Korea). Toluene (99.8%), tetrahydrofuran (THF, ≥99%), trifluorotoluene (TFT, ≥99%), and hexamethyldisilazane (≥99%) were purchased from Sigma Aldrich (St. Louis, MO, USA). Acetone (99.5%), ethanol (99.5%), and hydrochloric acid (37 wt%) were supplied by Samchun Chemical (Korea). All the chemicals were used as received without further purification.

### 2.2. Synthesis of FPSZs

FAS-17 (2 g, 3.52 mmol) was dissolved in a mixture of THF (10 g, 139 mmol) and deionized water (63.4 μL, 3.52 mmol). After stirring for 20 min at 25 °C, 8 g of OPSZ was added to the mixture. The mixture was then refluxed at 80 °C under continuous magnetic stirring for 24 h. Lastly, clear, pale yellow, low viscosity liquid FPSZs were obtained after the solvent and excess reagents were removed via distillation. The weight ratios of the fluorinated moieties of the FPSZ samples (x, wt%) were determined using ^1^H-nuclear magnetic resonance (NMR) analysis, and the FPSZ samples are denoted as FPSZ_x_. The fluorinated content of FPSZ was controlled by varying the FAS-17-to-OPSZ weight ratio, and the formulations are summarized in Table 1.

### 2.3. Fabrication of the Superhydrophobic Surfaces

Prior to polysilazane coating, the Al plates were etched to create a rough surface with a dual-scale structure. Etching was performed according to the procedure described in the literature [25]. First, the Al substrate was degreased ultrasonically using acetone and ethanol in sequence. Second, the cleaned Al plate was chemically etched in a 2.5 M HCl solution for 10 min at room temperature to construct the microstructure. After rinsing with deionized water, the substrate was immersed in boiling water for 30 min, and nanoscale structures were grown on the microstructure of the etched Al surface. Subsequently, the micro-nanostructure was dried at 120 °C for 2 h. The FPSZs were dissolved in a mixed TFT and toluene solvent with a TFT:toluene weight ratio of 1:3 (FPSZ in the coating solution of 7 wt%), followed by coating on the hierarchical Al plates via dip-coating for 30 min. The final superhydrophobic surfaces were obtained after drying the coated substrates at 150 °C for 24 h.

### 2.4. Characterizations and Water Repellent Tests

The grafting of fluorinated groups onto the polysilazane backbone was confirmed using the ^1^H-NMR spectra obtained using a 300 MHz (Bruker, Billerica, MA, USA) spectrometer in a CDCl_3_ solution. The Fourier-transform infrared (FTIR) spectra of the samples were obtained in the attenuated total reflection mode using a Nicolet 6700 (Thermo Scientific, Waltham, MA, USA) apparatus. The surface morphologies of the samples were evaluated using a field-emission scanning electron microscope (JSM-6701F, JEOL, Tokyo, Japan) at an acceleration voltage of 5 kV; the samples were sputter-coated with Pt prior to analysis. The chemical compositions of the coated surfaces were determined using an X-ray photoelectron spectroscopy (XPS; Thermo Scientific, Waltham, MA, USA) instrument with a monochromatic Al Kα X-ray source at a photon energy of 1486.7 eV. The CA and sliding angle (SA) of the coatings were measured using a Smart Drop (Femtobiomed, Seongnam, Korea) electronic device. A water droplet (8 µL) was placed on the coated surface at 25 °C, and the CA and SA were measured using the sessile drop method. The SA values were obtained by measuring the tilt angle of the coating where the water droplet slid off the surface at a tilt step of 0.2°. The reported CA and SA values are the averages of five measurements at different locations on each surface.

### 2.5. Anti-Icing Performance

#### 2.5.1. Icing-Delay Tests

Ice formation on the Al surface was evaluated using the crystallization temperature and freezing delay time of a water droplet on the surface. The crystallization temperature of a water droplet on each substrate was recorded using a differential scanning calorimetry (DSC; DSC 4000 Perkin Elmer, Foster City, CA, USA) instrument and a previously described method [26,27]. The coating surface was cut and placed into the sample pan (An Al sample pan has a length of 10 mm, a depth of 1.5 mm, and a volume of 30 μL), and then 5 μL of deionized water was added to the coated Al surface. Subsequently, the temperature was continuously lowered from room temperature to −25 °C at a cooling rate of 10 °C min^−1^. The crystallization temperature of each sample, which was defined as the onset freezing temperature, was determined using the corresponding exothermic peaks in the DSC curves. Furthermore, the crystallization temperature of the water droplets on each substrate was measured after 40 icing–melting cycles to determine coating durability. Freezing delay time was measured using a cooling chamber and a 50×–500× 8 LED USB digital microscope endoscope. Before measurement, each superhydrophobic surface was placed into a cooling chamber and cooled to −19.0 °C for 1 h to produce supercooled superhydrophobic surfaces in an unsaturated environment (70% relative humidity (RH)). Thereafter, a water droplet (10 μL) was placed on the sample surface. Subsequently, water droplet freezing was monitored and recorded using a camera. The freezing delay time was defined as the time at which the water droplet started clouding.

#### 2.5.2. Ice Adhesion Test

**Figure 1:**
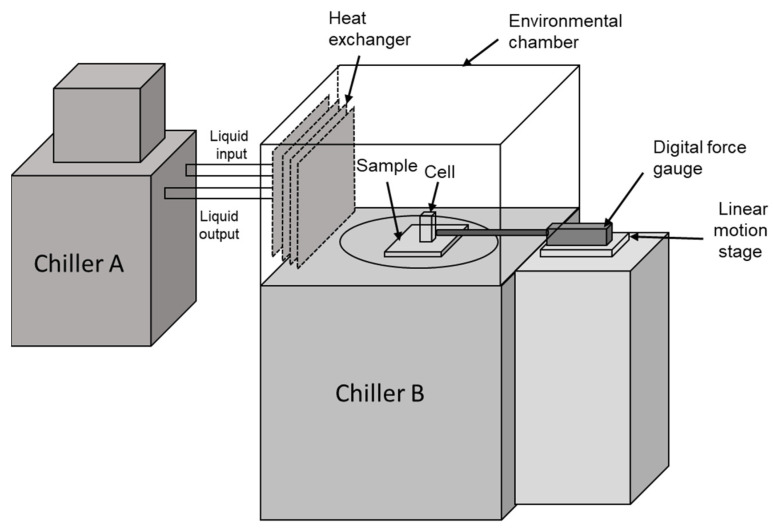
Schematic of the cooling chamber used for ice adhesion strength measurements.

In this study, we designed and constructed an ice adhesion testing apparatus using the information reported in several papers because instruments designed to measure ice adhesion strength are not commercially available [28,29]. The schematic of the designed instrument is presented in Figure 1. The internal dimensions of the environmental chamber were 320 mm wide × 350 mm long × 320 mm high. The temperature of the sample stage was controlled using chiller B, and the temperature of the free space inside the chamber was controlled using chiller A. The measurement procedure for each sample consisted of the following steps. A bottomless cell (1 cm × 1 cm × 3 cm) was placed on the sample stage, and then 1 mL of deionized water was filled in it. The water inside the cell was frozen at −20 °C for 3 h (70% RH). The temperature and RH inside the measurement system were recorded using a TR-72wf (T&D Corporation, Matsumoto, Japan) temperature/humidity recorder with relative errors of 0.3 °C and 2.5% RH, respectively. After freezing, the ice adhesion strength was measured by propelling the force probe perpendicularly to the ice column using a ZP 44 (Imada) force transducer with a 0.8 cm diameter probe at a predetermined speed and determining the shear stress required to detach the ice column from the surface. The measured maximum force at break was converted into ice adhesion strength, which was calculated using the equation *τ* = *F*/*A*, where *τ* is the ice adhesion strength, *F* is the critical force, and *A* is the contact area of the ice–substrate interface. The final values were determined by averaging the data from at least five replicates. The samples were subjected to multiple icing–melting tests using the same method as that for the ice adhesion strength tests. A water column, which was frozen at −20 °C for 3 h, was warmed to room temperature to melt the ice, followed by drying at 120 °C for 2 h for the next testing cycle. After 40 icing–melting cycles, the ice adhesion strength of each sample was measured to examine the durability of the substrate.

## 3. Results and Discussion

### 3.1. Synthesis and Identification of the FPSZs

FPSZs with low surface energy were synthesized via hydrolysis–condensation reactions of OPSZ with FAS-17. The typical FPSZ synthesis reactions are presented in Scheme 1B, and the synthesis steps were as follows. The methylhydrogensilane moieties (CH_3_–Si–H) of OPSZ reacted rapidly with a small amount of water to form silanol groups (CH_3_–Si–OH). The reactions involving the CH_3_–Si–OH groups proceeded via two primary pathways: (1) hydrolysis–condensation with the methoxysilane groups of FAS-17 and (2) condensation of the CH_3_–Si–OH groups. For reaction pathway (2), the cross-linking of the OPSZ chains increased its molecular weight, and then, the OPSZ chains reacted with FAS-17 to form an FPSZ with a basic structure ((3) in Scheme 1B).

**Figure 2:**
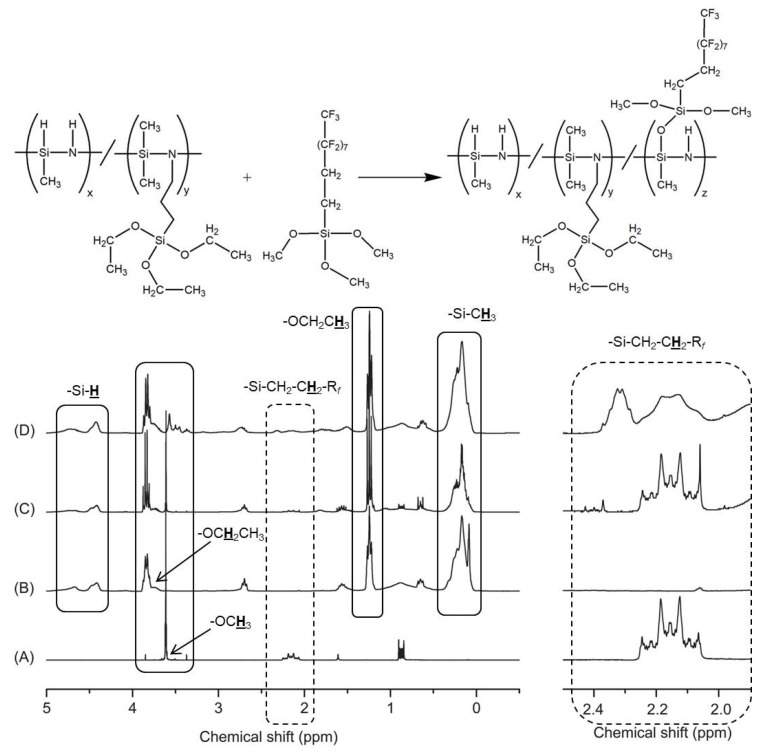
^1^H-NMR spectra of (**A**) FAS-17, (**B**) OPSZ, (**C**) the initial mixture of the reactants, and (**D**) FPSZ_17.3_.

The ^1^H-NMR spectra of FAS-17, OPSZ, initial mixture of the reactants, and FPSZ_17.3_ (Figure 2) confirmed the formation of FPSZs via the reaction of FAS-17 with OPSZ. Moreover, the spectra were used to determine the chemical structures and content of fluorinated moieties of the fabricated FPSZs. The ^1^H-NMR spectra of the OPSZ and FAS-17 mixture (Figure 2C) was compared with that of FPSZ_17.3_ (Figure 2D). The characteristic peaks in the ^1^H-NMR spectrum of the OPSZ and FAS-17 mixture matched those in the spectra of FAS-17 and OPSZ (Figure 2A,B, respectively). No significant changes were observed in the positions of the characteristic peak of the trimethoxy group (–O–CH_3_, 3.6 ppm) of FAS-17 and those of the hydrogen silane (Si–H, 4.35–5 ppm) and triethoxy groups (–O–CH_2_–CH_3_, 3.84 ppm and –O–CH_2_–CH_3_, 1.24 ppm) of OPSZ.

However, the positions of the characteristic peaks of the –O–CH_3_ and methylene (CH_2_–CF_2_) peaks in the ^1^H-NMR spectrum of FPSZ_17.3_ were shifted compared with those of the corresponding peaks in the ^1^H-NMR spectrum of FAS-17. The single –O–CH_3_ peak in the ^1^H-NMR spectrum of FAS-17 was split over the range of 3.45–3.6 ppm in the ^1^H-NMR spectrum of FPSZ_17.3_. The peak of CH_2_–CF_2_ was broadened and shifted to the left. The integration ratio of the CH_2_–CF_2_ and –O–CH_3_ peaks of 2:9 in the spectrum of FAS-17 was changed to 2:4.3 in the spectrum of FPSZ, indicating that half of the –O–CH_3_ groups participated in the reaction. In addition, the integral value of Si–H was lower than that of –Si–CH_3_ in the CH_3_–Si–H groups of FPSZ. Moreover, no significant changes were observed in the peak positions of the water-hydrolyzable N–H and triethoxysilane groups. These changes in peak positions indicated that hydrolysis and condensation with a small amount of water were the primary reactions between the CH_3_–Si–H groups of OPSZ and the trimethoxy groups of FAS-17. Therefore, FAS-17 was introduced into the backbone of OPSZ via hydrolysis and condensation reactions.

The weight contents of the fluoroalkyl (R*_f_*) moieties of the FPSZs were calculated by comparing the integrals of the –CH_2_–CF_2_ (2.1 ppm) and –Si–CH_3_ (0.1 ppm) peaks in the ^1^H-NMR spectrum of FPSZ_17.3_ (Figure 2D), and the results are summarized in Table 1. The proportion of R*_f_* moieties grafted onto OPSZ increased with increasing FAS-17 feeding ratio, indicating that the R*_f_* content of FPSZ can be well controlled by adjusting the FAS-17 feeding ratio. The conversion rates of all the reforming reactions were confirmed to be higher than 80%, indicating that this was a suitable method for introducing R*_f_* moieties into the OPSZ backbone.

### 3.2. Fabrication of Superhydrophobic Al Surface with Hierarchical Micro-Nanostructure

**Figure 3:**
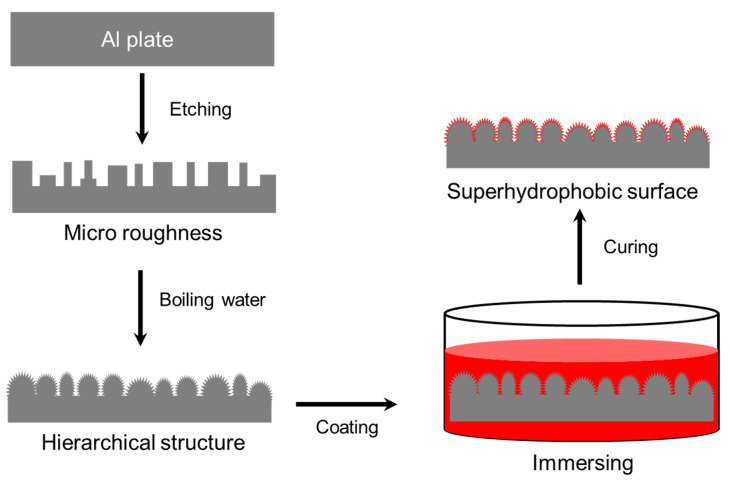
Schematic of the fabrication of superhydrophobic Al surfaces with micro-nanostructure.

The synthesized FPSZs were coated onto Al surfaces with micro-nano hierarchical structures to fabricate superhydrophobic surfaces. The typical method for fabricating superhydrophobic Al surfaces with hierarchical structures is presented in Figure 3. To obtain hierarchical structures on the Al surface, Al plates were etched with a HCl solution to generate a rectangular-shaped micro-roughness. Next, the micro-rough Al plates were immersed in boiling water, and nanostructures grew on the surfaces to obtain the final micro-nano hierarchical structure. Subsequently, the hierarchically rough Al plates were immersed in FPSZ dilute solutions, followed by drying to obtain superhydrophobic Al surfaces.

**Figure 4:**
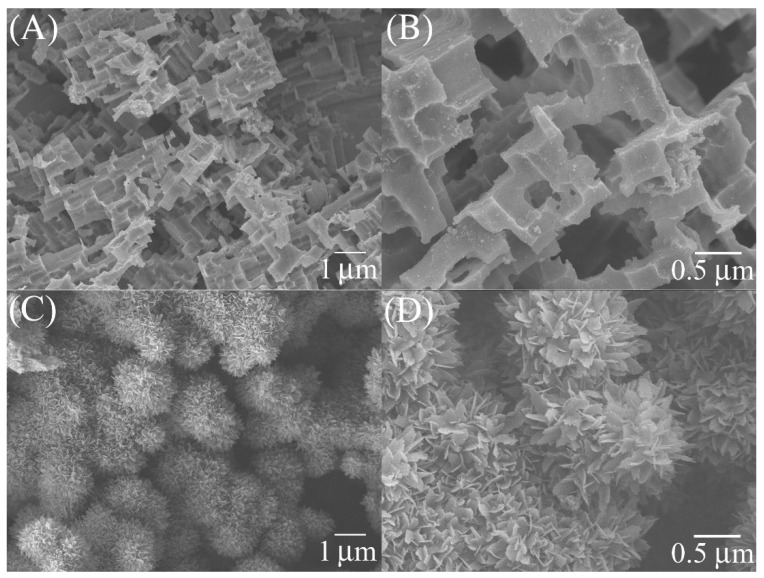
SEM images of the rough Al surfaces prepared via (**A**,**B**) HCl etching and (**C**,**D**) HCl etching followed by immersion in boiling water.

The scanning electron microscopy (SEM) images of the micro-nanostructured Al surfaces are presented in Figure 4. After etching with the HCl solution, the Al surface was rough and comprised rectangular-shaped microscale patterns with lengths in the range of 0.2–1.0 μm (Figure 4A,B). This microstructure was formed because of selective etching of vulnerable dislocation inside the Al crystals [30] Upon immersing the etched plate with a microscale pattern in boiling water, the Al surface reacted with water to form crystalline boehmite (AlO(OH)), resulting in a nano-flake structure [25,31]. The SEM images of the Al surface after immersion in boiling water (Figure 4C,D) revealed that a 20 nm thick × 200 nm long flower petal-shaped nanostructure was well formed and densely distributed on individual microscale Al spheres. The superhydrophilicity of the hierarchical micro-nano structure was attributed to the hydrophilicity of the surface, and a material with a low surface energy was required to convert this surface into a superhydrophobic surface. FPSZs were primarily fabricated as coating materials to enhance the superhydrophobicity and anti-icing properties of Al surfaces. To evaluate the properties of the fabricated FPSZs, hierarchically micro-nanostructured Al samples were coated with FPSZs using an immersion method.

After the hierarchical micro-nano structured Al plates were coated with FPSZs, they were subjected to water–oil wettability measurements, and the results are summarized in Table 1. To compare the effect of fluorinated functional groups, unmodified OPSZ and OPSZ–FAS-17 mixtures were also coated on hierarchical Al plates, and the water wettability of these surfaces was evaluated. The water CA/SA values on the OPSZ- and OPSZ–FAS-17 mixture-coated surfaces were 167°/4° and 169°/7°, respectively. The superhydrophobicity of the OPSZ-coated surface was ascribed to the methyl groups in the backbone, and the distribution of the CA/SA values was even over the entire measurement area. For the oil CA measurements using hexadecane, the OPSZ-coated surface was wetted because of the presence of the lipophilic methyl groups of OPSZ. Furthermore, the water CA of the OPSZ–FAS-17 mixture-coated surface was higher than that of the OPSZ-coated surface; however, the sliding properties of the OPSZ–FAS-17 mixture-coated surface were inferior to those of the OPSZ-coated surface. Moreover, the error ranges of the CA and SA of the OPSZ–FAS-17 mixture-coated surface were three and eight times larger, respectively, than those of the OPSZ-coated surface. This indicated that the OPSZ–FAS-17 mixture did not form an even coating over the entire measurement area because both OPSZ and FAS-17 formed Al–O–Si bonds [32] with the Al surface. The OPSZ layer did not form easily in the areas where amphiphobic FAS-17 reacted first with the Al surface; therefore, the Al surface was not evenly coated. Upon placing hexadecane drops onto the analyzed surfaces, hexadecane wetted most of the surface; however, the oil droplets presented CAs of approximately 50° over small areas.

The errors ranges of the CAs and SAs of the FPSZ-coated surfaces were smaller than those of the OPSZ–FAS-17 mixture-coated surface. In addition, the water CAs and SAs of the FPSZ-coated surfaces increased and gradually decreased, respectively, by increasing the content of R*_f_* moieties introduced into the OPSZ backbone. Because the surface energies of fluorocarbons comprising –CF_3_ and –CF_2_ functional groups are lower than those of the corresponding hydrocarbons, increasing the fluorinated content of FPSZ increased the hydrophobicity of the coated surface. When the R*_f_* content of FPSZ reached 17.3 wt%, the water CA and SA of the coated surface increased to 175° and decreased to 1.6°, respectively, and the CA and SA values were unchanged even at higher R*_f_* contents. Moreover, the number of Si–H functional groups of the FPSZ backbone was decreased by the introduction of excess R*_f_* moieties, which hindered the formation of a dense coating layer. As for the oil CA, hexadecane wetted the surfaces of the samples coated with FPSZs with R*_f_* contents of up to 12.7 wt%, and the oil CA was 50° for the FPSZ_17.3_-coated sample. The dependence of the oil CA on the R*_f_* content was similar to that reported in our previous paper; however, the method used to introduce R*_f_* moieties into the backbone of OPSZ in this study was simpler [21].

### 3.3. Anti-Icing Properties

**Figure 5:**
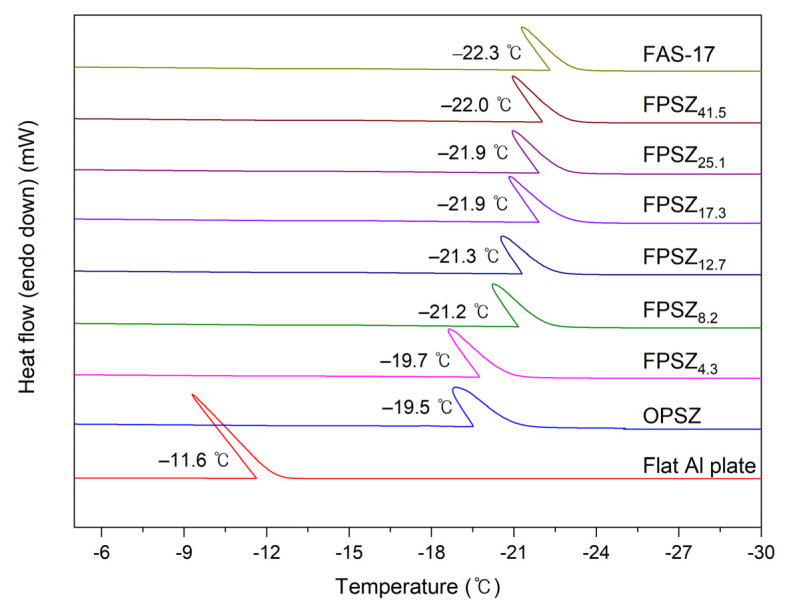
DSC curves of super-cooled water droplets dripped on the OPSZ-, FPSZ-, and FAS-17-coated rough Al surfaces. A flat Al substrate was used for comparison. The temperature was set to decrease at a rate of 10 °C min^−1^.

The primary parameters for evaluating the anti-icing performance of the fabricated surfaces were the ice nucleation rate and ice adhesion strength. To exhibit excellent anti-icing properties, surfaces must present low ice adhesion strength and excellent ability to inhibit ice nucleation [33,34]. In our previous study, we confirmed that FPSZs are good anti-icing materials [21]. The ice nucleation rates of the FPSZ-coated Al surfaces in this study indicated that the FPSZs fabricated herein presented remarkable potential as anti-icing materials (Figure 5). Improvement in icephobicity was expected with increasing the R*_f_* content of FPSZs, which was confirmed by the delays in condensed water crystallization [27]. The changes in the crystallization temperature of water droplets were determined using the DSC curves obtained by continuously lowering the temperature, and the crystallization temperature was defined as the onset temperature of the freezing peak. When water freezes, latent heat is released and the temperature of the droplet is raised, resulting in the formation of the slanted peaks. According to the DSC curves (Figure 5), the freezing temperatures of water droplets on the superhydrophobic OPSZ- and FPSZ-coated surfaces were lower than −19.5 °C, whereas that on a flat Al surface was −11.6 °C. Because abundant air pockets formed between water droplets and the micro-nano structured Al surfaces coated with low-surface-energy materials, heat transfer and ice formation were delayed. As the R*_f_* content of FPSZ was increased, the freezing point of the water droplets gradually decreased and reached the lowest value of −22.0 °C for FPSZ_41.5_. In addition, the freezing temperature of FAS-17, with an R*_f_* group content of 100 wt%, was the lowest among all the samples (−22.3 °C). The decrease in the freezing temperature of water with increasing R*_f_* content was consistent with the increase in the water/oil Cas. The freezing temperature of water on the FPSZ-coated surfaces decreased with increasing R*_f_* content because the surface energy of the R*_f_* group was significantly lower than that of the methyl groups of OPSZ. Therefore, the contact area between water and the coating layer was minimized, and heat transfer was reduced. Moreover, crystallization times were calculated when the DSC curves in Figure 5 was plotted as a function of time. These are in the range of 24 to 33 s, but any consistent pattern was not found for all samples.

**Figure 6:**
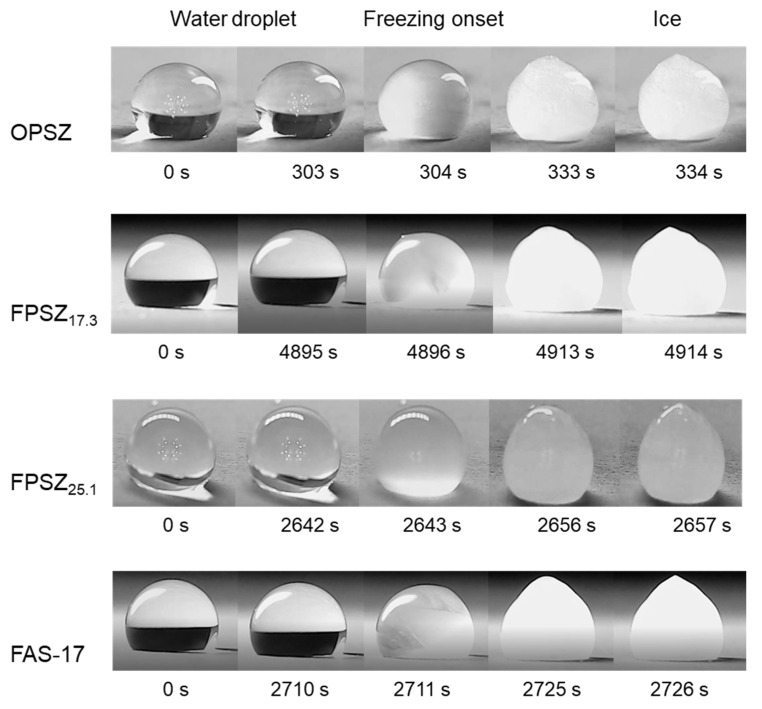
Optical images of 10 μL water droplets on coated Al surfaces.

DSC is a useful method for comparing the ice-forming temperatures of superhydrophobic surfaces under low RH conditions because measurements are performed with a nitrogen atmosphere. However, as the RH increases, the wettability of some superhydrophobic surfaces changes to a “Wenzel state,” which deteriorates the anti-icing performance of the surfaces [27]. The freezing of water droplets on the surfaces at 70 ± 5% RH and −19.0 °C was recorded, and images were captured at different times (Figure 6). The freezing times of water droplets on the OPSZ-, FPSZ_17.3_-, FPSZ_25.1_-, and FAS-17–coated Al surfaces were 334, 4914, 2657, and 2726 s, respectively. The freezing delay time of the water droplets on the FPSZ_17.3_-coated Al surface was the longest. The freezing delay time of the FAS-17-coated Al surface was significantly shorter than that of the FPSZ_17.3_-coated Al surface and was similar to that of FPSZ_25.1_-coated Al surface. These results were closely related to the increase in ice adhesion strength on the surface of the FAS-17-coated Al with increasing RH reported in our previous paper [27]. The substrate–liquid interface of the superhydrophobic surface changed from the “Cassie state” to the Wenzel state because of the increase in RH; moreover, the contact area was widened. The FPSZ_17.3_-coated surface presented the best freezing delay effect because the change in solid–liquid interface with RH was negligible compared with those of the other surfaces.

**Figure 7:**
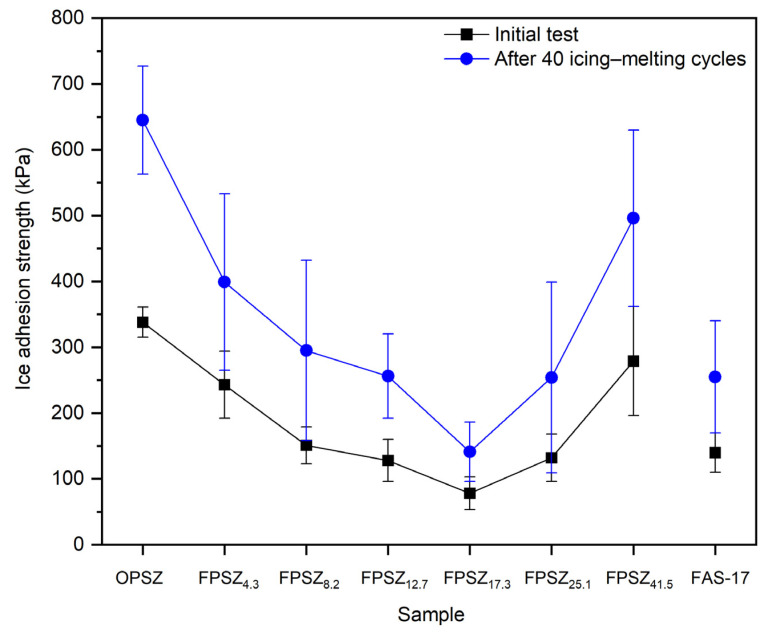
Ice adhesion strengths of the OPSZ-, FPSZ-, and FAS-17-coated rough Al surfaces before and after 40 icing–melting cycles.

The freezing delay time indicates how long ice nucleation is delayed at the water–solid interface. The anti-icing properties of surfaces can also be evaluated using ice adhesion strength measurements. Ice adhesion strength measurements consist of directly measuring the force required to remove ice frozen on a solid surface, and low ice adhesion strength values translate into excellent anti-icing properties. The ice adhesion strengths of the OPSZ- and FPSZ-coated Al plates are presented in Figure 7. The initial ice adhesion strengths of the FPSZ-coated Al plates were lower than that of the OPSZ-coated Al plate. Although the OPSZ- and FPSZ-coated Al plates presented superhydrophobic properties in wettability tests, the ice adhesion strengths of the FPSZs were relatively superior to that of the OPSZ-coated Al plate because of the low surface energy of the R*_f_* groups, which significantly affected ice adhesion strength [21]. Ice adhesion strength decreased with increasing R*_f_* content up to 17.3 wt%; however, it increased significantly at R*_f_* contents higher than 17.3 wt%. This was attributed to the decrease in solubility of FPSZs with high R*_f_* content and the decrease in coating–substrate compatibility. Furthermore, the ice adhesion strength of the FAS-17-coated Al plate with the highest R*_f_* content (100%) was 140 kPa, which was higher than that of the FPSZ_17.3_-coated Al plate (78 kPa). As the R*_f_* content was increased, a dense R*_f_* layer formed at the air–solid interface. The dense layer prevented the effective exposure of the surface Al–OH groups to water vapor, and the effect was maximized at an R*_f_* content of 17.3 wt%. However, at R*_f_* contents >17.3 wt%, the number of Si–H functional groups of FPSZ capable of self-condensation was relatively low; therefore, the coating layer became thinner. In addition, the FPSZs with extremely high R*_f_* contents presented low solubility and high adhesion to the substrate [21]. This led to the formation of a thin, non-uniform coating layer, which deteriorated the icephobicity of the coating and presented a wide error range. Unlike FPSZs, FAS-17 formed a uniform monolayer coating; however, the coating layer was thin. Consequently, the ice adhesion strength of the FAS-17-coated surface was intermediate compared with those of the FPSZ-coated surfaces. To obtain an FPSZ-coated superhydrophobic surface with low ice adhesion, abundant air pockets should form under the water droplets, and the contact surface area of the water droplets with the nanostructured surface should be minimized. Upon compressing the air pockets trapped in structures, they act as stress concentrators and exert a counter force on the ice column, leading to a decrease in ice adhesion strength [15,16]. The air pockets minimize the contact surface area between the ice and the substrate even during water freezing. Superhydrophobic Al surfaces can become mechanically damaged, and the middle of the ice column on the surface can be broken if the number of the air pockets at the interface is insufficient because of incomplete coating coverage or the lack of a dense coating layer [12,35]. In other words, water vapor that diffused into the air pockets during ice formation condensed at the hydrophilic sites of the imperfect superhydrophobic surface; therefore, the water droplets formed “Wenzel ice” [14,17]. To minimize the formation of Wenzel ice, the interactions between the hydrophilic Al–OH groups at the Al surface and water should be minimized by maintaining sufficient distance between them. Thick coating layers appear to minimize these interactions; however, the hierarchical nanostructured pores can be filled with coating material, which suppresses superhydrophobicity. Thickness control at the molecular level is required to simultaneously achieve a sufficiently thick coating layer and superhydrophobicity.

The surface energy of the R*_f_* groups of FPSZs is lower than that of the CH_3_–Si groups of OPSZ and the R*_f_* chains arrange densely at the solid–air interface above a certain R*_f_* content [36,37]. The thickness of the coating layer increased at the molecular level because of the surface orientation of the R*_f_* groups. The XPS data in Table 2 indicate the surface-covering effect caused by the orientation of the R*_f_* groups after FPSZ coating. XPS analysis can be used to identify the atoms present in a 10 nm thick layer beneath the surface. Our results indicated that the amount of Al in the substrate decreased as the thickness of the coating layer increased. The XPS-determined Al contents of the OPSZ- and FPSZ_17.3_-coated Al samples were 16.8% and 1.4%, respectively. By contrast, the Al content of the FAS-17-coated Al sample was 27.6%, indicating that the FPSZ_17.3_ coating layer was thicker than the OPSZ and FAS-17 coating monolayers. In addition, as the R*_f_* content of the FPSZ layer increased, the Al 2p values were decreased until the R*_f_* reached 17.3 and were then increased at contents higher than 17.3. The coating layers of FPSZs with high R*_f_* contents (>17.3) were relatively non-uniform compared to that of FPSZ_17.3_, and non-uniformity was closely related to the rapid increase in ice adhesion strength.

### 3.4. Durability of the Anti-Icing Surfaces

**Figure 8:**
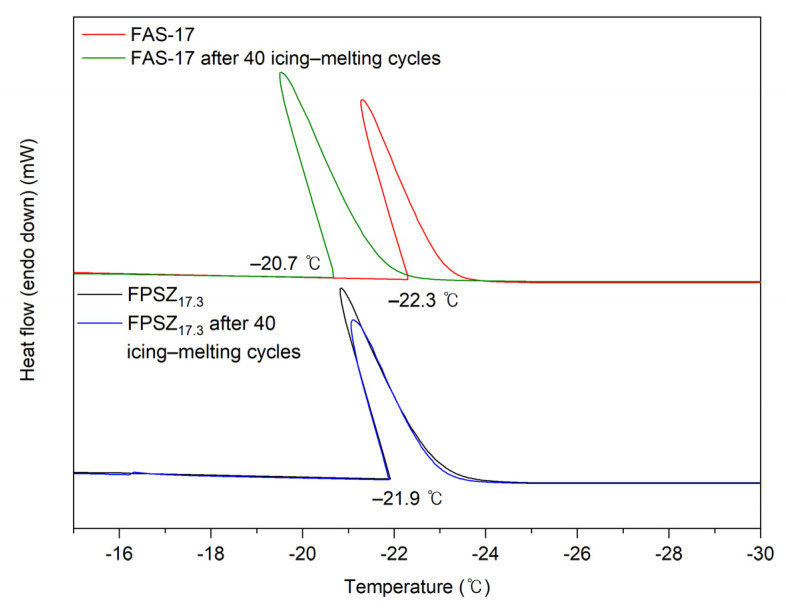
DSC curves of supercooled water droplets dropped on the FPSZ_17.3_- and FAS-17–coated Al surfaces before and after 40 icing–melting cycles.

Superhydrophobic surfaces exhibit low ice adhesion strength and delay the freezing of water droplets on them; however, surface durability is not proportional with surface wettability (CA and SA) or initial ice adhesion strength. A hierarchically rough surface is typically weak under physical impact because of the fragility of the micro-nano surface structure. During freezing, water expansion against the substrate causes damage to the hierarchical structure such that ice adhesion strength increases and surface durability deteriorates. Therefore, it is critical to improve the mechanical stability and durability of superhydrophobic surfaces for practical applications. In this study, the robustness and durability of the superhydrophobic surfaces were studied by evaluating the ice adhesion strength of the icephobic coatings after multiple icing–melting cycles (Figure 7). The adhesion between the OPSZ-coated surface and the ice column was remarkably strong. After 40 icing–melting cycles the ice column broke, and ice fragments remained on the surface. The damage to the OPSZ-coated surface and loss of icephobic properties after multiple icing–melting cycles were associated with the change in wetting state that occurred when the water in the column froze [38]. During repeating icing–melting cycles, the water vapor inside the surface pores underwent diffusion and condensation repeatedly, causing the wettability state to gradually change from Cassie–Baxter to Wenzel [6]. When water freezes in the Wenzel state, the volume of Wenzel water expands inside the pores. The force generated via this volume expansion causes structural damage to the superhydrophobic surface, resulting in a sharp increase in ice adhesion strength after multiple icing–melting cycles. To improve the durability of superhydrophobic surfaces, it is critical to prevent water from penetrating and condensing into the surface pores during icing–melting cycles. In addition, the structure should present adequate strength to withstand the force caused by the volume expansion that occurs during ice formation. The ice-adhesion strength of the FPSZ-coated samples increased after 40 icing–melting cycles, and intermittent cohesive failures were observed for the FPSZ_4.3_- and FPSZ_41.5_-coated samples. Conversely, the ice adhesion strength of the FPSZ_17.3_-coated sample after 40 icing–melting cycles (141 kPa) was 80.7% higher than the initial value. The ice adhesion strengths of the FPSZ_8.2_-, FPSZ_12.7_-, FPSZ_25.1_-, and FAS-17-coated samples after 40 icing–melting cycles were 95.3%, 100%, 92.4%, and 82.0% higher, respectively, than the initial values. Moreover, these values were higher than that of the FPSZ_17.3_-coated sample. Therefore, the surface durabilities of these samples were inferior to that of the FPSZ_17.3_-coated sample. Consequently, we concluded that FPSZ_17.3_ was a suitable coating material, featuring a good balance between the excellent hardness of OPSZ and the low surface energy of FAS-17. Unlike the FAS-17 monolayer coating, the relatively thick FPSZ_17.3_ coating layer can protect the Al surface from direct physical impact owing to its excellent mechanical properties. FPSZ_17.3_, which is a polysilazane-based material, formed a hard coating layer through self-condensation and formed chemical bonds with the Al substrate surface. The hard coating physically protected the nanostructure of the physically weak superhydrophobic Al surface. As expected, the nanoscale structure of the FAS-17 coating (Figure S1) was almost completely damaged after sand impact, whereas the structure of the FPSZ_17.3_ coating was less damaged. Unlike the durability of the FAS-17 coating, that of the FPSZ_17.3_ coating was improved after 40 icing–melting cycles. The initial ice formation temperature of FAS-17 (−22.3 °C) increased to −20.7 °C after 40 icing–melting cycles (Figure 8). The freezing temperature of water droplets on the FPSZ_17.3_-coated surface after 40 icing–melting cycles was almost unchanged, indicating the superior durability of FPSZ_17.3_ relative to that of FAS-17. The surface coated with FPSZ_17.3_ showed anti-icing performance comparable to other systems described in the literature [19,20].

## 4. Conclusions

Superhydrophobic surfaces with excellent icephobicity and durability were prepared by coating micro-nanostructured Al substrates with FPSZs. To provide low surface energy and physical stability, fluorinated segments (FAS-17) were introduced into an OPSZ backbone. The fabricated FPSZs improved the icephobicity and durability of the rough Al substrates, which were pretreated using an HCl solution and boiling water. Among the coating materials, FPSZ_17.3_ presented the optimal R*_f_* content, exhibiting the lowest ice adhesion strengths before and after multiple icing–melting cycles and the highest freezing delay time. The rough Al surface coated with fluorine-free OPSZ exhibited superhydrophobicity with a high-water CA (167°); however, its ice adhesion strength reached 338 kPa. The FAS-17-coated Al surface presented superomniphobicity, owing to the high content of fluorinated alkyl groups of FAS-17. However, the adhesion strength of the FAS-17-coated Al substrate was relatively high (140 kPa) compared to that of the FPSZ_17.3_-coated Al substrate (78 kPa). The V-shape of the plot of the ice adhesion strengths of the FPSZ-coated samples as a function of the R*_f_* content of the FPSZ layers was centered at an R*_f_* content of 17.3 wt%. The icephobicity results indicated that the Al substrates with remarkable anti-icing performance required an adequate fluoroalkyl content (low surface energy) and a suitable coating layer thickness. The anti-icing properties of the FPSZ_17.3_-coated surface were excellent even after 40 icing–melting cycles. The accumulated ice was easily removed from the surfaces with low ice adhesion strengths (78 kPa initially and 141 kPa after 40 icing–melting cycles). Moreover, the ice adhesion strengths of the FPSZ_17.3_-coated surface were lower than the initial ice adhesion strength of the OPSZ-coated Al surface (338 kPa). The low surface energy of the fluoroalkyl groups hindered the transition of the surface wetting state from the Cassie–Baxter to the Wenzel mode. In addition, the abundant air pockets at the solid–liquid interface of the FPSZ-coated surface served as thermal insulators. Therefore, the ice nucleation temperature of water droplets on the surface decreased to −21.9 °C, and the time required for ice to form was 4914 s at −19.0 °C because FPSZs suppressed ice nucleation of the water droplets. The long-term icephobicity of the FPSZ-coated surfaces was ascribed to the good coverage of fluorinated segments on the surface with adequate polymer coating thickness. Therefore, our results suggest that FPSZ superhydrophobic coatings can be potential passive anti-icing candidates for various applications. This study offers insight into the development of robust anti-icing surfaces for practical applications such as outdoor coil of air source heat pumps or aircrafts.

## Figures and Tables

**Table 1:** Wettability properties of the OPSZ-, FPSZ-, FAS-17–, and OPSZ–FAS-17 mixture-coated Al surfaces.

Samples	FAS-17 Feed Ratio (wt%)	FAS-17 Incorporated Ratio ^a^ (wt%)	Conversion (%)	Water		Oil CA ^b^ (°)
				CA (°)	SA (°)	
OPSZ	0			167 ± 1.2	4.0 ± 0.5	wetted
FPSZ_4.3_	5	4.3	86.0	169 ± 1.5	3.2 ± 0.4	
FPSZ_8.2_	10	8.2	82.0	170 ± 1.5	2.2 ± 0.3	
FPSZ_12.7_	15	12.7	84.6	172 ± 1.3	2.1 ± 0.3	
FPSZ_17.3_	20	17.3	86.5	175 ± 1.4	1.6 ± 0.2	50 ± 6.0
FPSZ_25.1_	30	25.1	83.7	175 ± 1.6	1.7 ± 0.2	73 ± 4.5
FPSZ_41.5_	50	41.5	83	175 ± 1.8	1.6 ± 0.2	83 ± 3.5
FAS-17	100			175 ± 1.7	1.5 ± 0.2	150 ± 3.0
OPSZ–FAS-17 ^c^	17.3	17.3	-	169 ± 3.5	7.0 ± 4.0	mostly wetted

^a^ Determined using 1H-NMR data. ^b^ Measured 5 min after hexadecane was dropped onto the sample surface. ^c^ OPSZ and FAS-17 mixture.

**Table 2:** Elemental compositions of the OPSZ-, FAS-17- and FPSZ-coated rough Al substrates.

XPS Atomic Concentration (%)								
Atom	OPSZ	FPSZ_4.3_	FPSZ_8.2_	FPSZ_12.7_	FPSZ_17.3_	FPSZ_25.1_	FPSZ_41.5_	FAS-17
Si 2p	21.3	26.4	23.0	19.7	15.2	14.5	12.5	0.8
C 1s	16.3	25.1	26.6	27.0	31.5	30.2	12.3	14.3
N 1s	0.9	1.2	1.0	1.5	1.2	1.5	0.5	0.1
O 1s	44.6	33.6	28.4	27.4	17.7	17.6	27.5	43.6
Al 2p	16.8	2.5 (2.8) ^a^	1.8 (2.2)	4.4 (5.5)	0.9 (1.4)	2.2 (3.3)	8.5 (13.9)	22.4 (27.6)
F 1s	0.0	11.2	19.2	20.0	33.5	34.4	38.7	18.8

^a^ The numbers in parentheses in the Al 2p row are the values excluding F.

## Data Availability

Not Applicable.

## Supplementary Materials

The following supporting information can be downloaded at: https://www.mdpi.com/article/10.3390/polym14020330/s1, Figure S1: SEM images of (A) FAS-17- and (B) FPSZ_17.3_-coated Al samples after sand impact.
