# Supplementary material for: Facile Synthesis of Fluorinated Polysilazanes and Their Durable Icephobicity on Rough Al Surfaces
Source: Polymers (Basel). 2022 Jan 14;14(2):330. doi: 10.3390/polym14020330 (PMC8779161; doi:10.3390/polym14020330)
Supplement: Supplementary file 1 [file polymers-14-00330-s001.zip › polymers-1525786-supplementary.pdf]

polymers

Supporting Information

Facile Synthesis of Fluorinated Polysilazanes and Their Durable Icephobicity on Rough Al Surfaces

Tien N. H. Lo,[1] Sung Woo Hong[1] and Ha Soo Hwang [2,*] In Park [1,3,*]

[1] Research Institute of Clean Manufacturing System, Korea Institute of Industrial Technology (KITECH), 89 Yangdaegiro-gil, Ipjang-myeon, Cheonan 31056, South Korea
[2] R&D center, OomphChem Inc., 1223-24 Cheonan-daero, Seobuk-gu, Cheonan, 31080, South Korea
[3] KITECH school, University of Science and Technology (UST), 176 Gajeong-dong, Yuseong-gu, Daejeon 34113, South Korea
* Correspondence: heliocity@naver.com (Ha Soo Hwang), inpark@kitech.re.kr (In Park)

Sand impact test

The mechanical durability of the superhydrophobic surfaces was evaluated and the durability of the FPSZ coatings on micro-nanostructured Al surfaces was compared with that of the FAS-17 coating. Sand impact tests were performed by dropping 10 g of sea sand (100–300 μm) onto the tilted substrate surface (tilt angle of 45°) for 1 min from a 30 cm height above the substrate [S1].

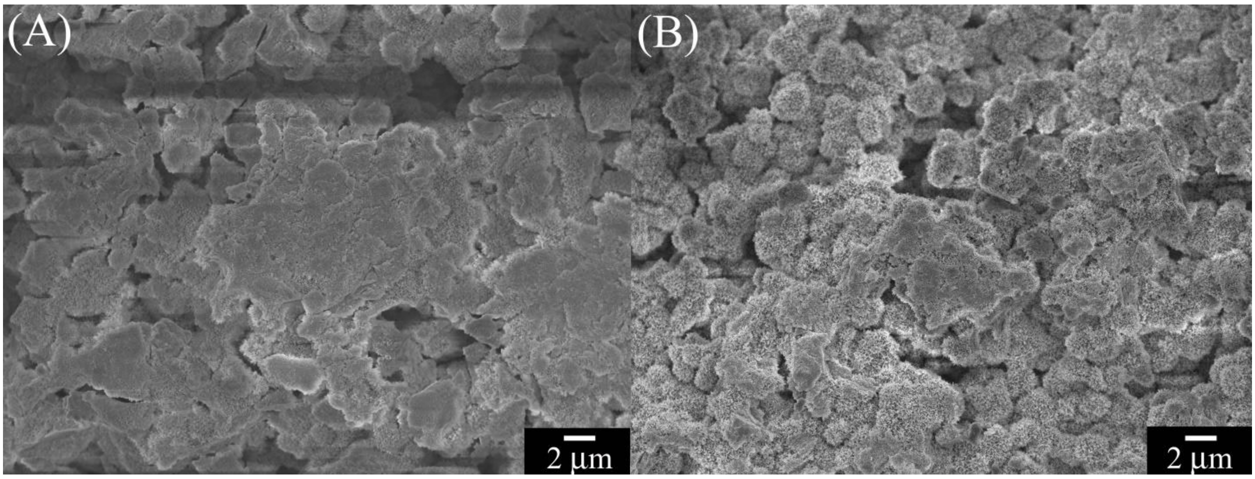

Figure S1. SEM images of (A) FAS-17– and (B) $FPSZ_{17.3}$-coated Al samples after sand impact.

Reference

[S1] Y. Zhang, D. Ge, S. Yang, Spray-coating of superhydrophobic aluminum alloys with enhanced mechanical robustness, J. Colloid Interface Sci. 423 (2014) 101–107. https://doi.org/10.1016/j.jcis.2014.02.024.
